# Genome Sequencing of Rare Disease Patients Through the Korean Regional Rare Disease Diagnostic Support Program

**DOI:** 10.1155/humu/6096758

**Published:** 2025-02-27

**Authors:** Rin Khang, Hane Lee, Jihye Kim, Dongseok Moon, Seokhui Jang, Eugene Lee, Yongjun Song, Seung Woo Ryu, Sohyun Lee, Heonjong Han, Sukwon Kim, Sohyun Jang, Young Bae Sohn, Won Seop Kim, Ji-Eun Lee, Juwon Kim, Yonggon Cho, Bo Lyun Lee, Han Hyuk Lim, Hoon Kook, Ki-Soo Kang, Soonhak Kwon, Jiwon Lee, Go Hun Seo, Seung Hwan Oh, Chong Kun Cheon

**Affiliations:** ^1^Medical Genetics Division, 3billion Inc., Seoul, Republic of Korea; ^2^Research and Development Center, 3billion Inc., Seoul, Republic of Korea; ^3^Rare Disease Center of Southern Gyeonggi Region, Department of Medical Genetics, Ajou University Hospital, Ajou University School of Medicine, Suwon, Republic of Korea; ^4^Rare Disease Center of Chungbuk Region, Department of Pediatrics, Chungbuk National University Hospital, Cheongju, Republic of Korea; ^5^Rare Disease Center of Northwestern Gyeonggi Province, Department of Pediatrics, Inha University Hospital, Incheon, Republic of Korea; ^6^Rare Disease Center of Gangwon Region, Yonsei University Wonju College of Medicine, Wonju Severance Christian Hospital, Wonju, Republic of Korea; ^7^Jeonbuk Regional Center for Rare Diseases, Department of Laboratory Medicine, Jeonbuk National University Hospital, Jeonju, Republic of Korea; ^8^Rare Disease Center of Busan Region, Department of Pediatrics, Busan Paik Hospital, Inje University College of Medicine, Busan, Republic of Korea; ^9^Rare Disease Center of Chungnam Region, Department of Pediatrics, Chungnam National University Hospital, Chungnam National University College of Medicine, Daejeon, Republic of Korea; ^10^Rare Disease Center of Chonnam Region, Department of Pediatrics, Chonnam National University Hwasun Hospital, Gwangju, Republic of Korea; ^11^Rare Disease Center of Jeju Region, Department of Pediatrics, Jeju National University Hospital, Jeju National University College of Medicine, Jeju, Republic of Korea; ^12^Rare Disease Center for Daegu/Gyeongbuk Region and Department of Pediatrics, Kyungpook National University Children's Hospital and School of Medicine, Kyungpook National University, Daegu, Republic of Korea; ^13^Division of Rare Disease Management, Korea Disease Control and Prevention Agency, Cheongju, Republic of Korea; ^14^Department of Laboratory Medicine, Pusan National University Yangsan Hospital, Pusan National University School of Medicine, Yangsan, Republic of Korea; ^15^Rare Disease Center of Gyeongnam Region, Department of Pediatrics, Pusan National University Children's Hospital, Pusan National University School of Medicine, Yangsan, Republic of Korea

## Abstract

Affecting fewer than 20,000 people as defined in South Korea, rare diseases pose significant diagnostic challenges due to their diverse manifestations and genetic heterogeneity. Genome sequencing (GS) offers a promising solution by enabling simultaneous screening for thousands of rare genetic disorders. This study explores the diagnostic utility and necessity of GS within the government-funded Korean Regional Rare Disease Diagnostic Support Program (KR-RDSP), a collaborative initiative involving 11 regional rare disease centers across Korea. The program was launched as a proof-of-concept study in 2023 to equip the genetic clinics with a diagnostic tool to expedite the diagnoses for rare disease patients who reside outside the urban Seoul region where diagnostic resources are limited. The study leveraged GS to diagnose a cohort of 400 patients exhibiting a wide spectrum of symptoms. The overall diagnostic yield was 36.3% (145/400), with 4.8% (7/145) of the diagnosed patients being reported with variants that could not have been identified by chromosomal microarray or exome sequencing (ES), highlighting the added value of comprehensive genomic analysis. The implementation of a centralized GS analysis system streamlined the diagnostic process, enabling timely reporting within a reasonable turnaround time of ≤ 35 days. Segregation analysis by Sanger sequencing played a crucial role in confirming or reclassifying variant pathogenicity by elucidating inheritance patterns. Here, we summarize diagnostic statistics from the 400 GS dataset gathered from June 2023 to December 2023 and show interesting and informative case examples that illustrate the diagnostic efficacy of GS, highlighting its ability to uncover elusive genetic etiologies and provide personalized treatment insights. The study also highlights the successful implementation of the program for the 11 regional rare disease centers across Korea with a practical workflow, comprehensive testing, comparable diagnostic yield to previous reports, and, most importantly, reasonable turnaround time.

## 1. Introduction

In the Republic of Korea (Korea), under the Rare Disease Management Act [1], a rare disease is defined as affecting fewer than 20,000 people or having an unknown carrier count due to diagnostic challenges, as determined in accordance with the procedures and standards prescribed by the Ordinance of the Ministry of Health and Welfare. With a population nearing 52 million [2], over 50,000 new rare disease patients are clinically diagnosed annually [3]. Since 2021, 12 regional centers for rare diseases have been designated in major hospitals in Seoul and other metropolitan areas/provinces to carry out rare disease diagnosis support programs [4]. However, with the greater Seoul population exceeding 50% of Korea [5], the number of patients in the regional area is proportionally lower. Therefore, it is not efficient for each hospital to establish genomic testing infrastructure, and eventually, patients in the region would be transferred to a hospital in Seoul with the infrastructure to receive a genetic diagnosis. However, travel can be a major challenge for many rare disease patients and their families [6].

Last year, the Korea Disease Control and Prevention Agency (KDCA) established the first pilot study, the Korean Regional Rare Disease Diagnostic Support Program (KR-RDSP), to investigate whether the diagnostic odyssey could be made more efficient with reduced turnaround time (TAT) for patients living in regional areas by providing means to receive genetic testing through their regional rare disease centers in Korea. Genome sequencing (GS) was chosen as the test type because it is the most comprehensive test that can identify additional types of variants that exome sequencing (ES) or chromosomal microarray (CMA) cannot detect and thus has higher clinical utility [7–12]. In the most recent study, 8.2% of all patients (61/744) were diagnosed with variants that required GS for identification [12]. GS is also readily available at a reasonable cost [13]. GS would be performed at a single reference laboratory in Seoul, but patients would not have to travel to receive the test. Here, we summarize the outcomes of this pilot study.

## 2. Materials and Methods

### 2.1. Referrals and Sample Collection

The KR-RDSP was supported by the Division of Rare Disease Management of the KDCA from April to December 2023. Led by Pusan National University Yangsan Hospital, 11 rare disease regional centers from 11 metropolitan areas/provinces participated. The KDCA established a centralized, secure online portal to order and receive test results. Each center screened for undiagnosed patients likely to have rare genetic diseases. Whole blood samples were collected in ethylenediaminetetraacetic acid (EDTA) tubes, except for one patient who provided DBS from heel pricking. Samples were transported to the reference laboratory for GS by courier service within 2 days of collection (Figure S1). The TAT was defined as the period from the date of sample reception to the date the GS report was released.

### 2.2. GS and Variant Calling

High molecular weight genomic DNA was extracted from the specimens using the QIAamp DNA Blood Mini Kit (Qiagen, Hilden, Germany). The sequencing library was prepared using the TruSeq DNA PCR-Free kit (Illumina, San Diego, CA, United States), and sequencing was performed using NovaSeq 6000 (Illumina) as 150 bp paired-end sequencing.

Sequencing data analysis was performed using a bioinformatics pipeline built with various publicly available tools (Figure S2). The binary base call (BCL) files were converted to FASTQ files using bcl2fastq (v2.20.0.422; Illumina, San Diego, CA, United States). Sequence reads were aligned to the Genome Reference Consortium Human Build 38 (GRCh38) [14] and the Revised Cambridge Reference Sequence (rCRS) [15] of the mitochondrial genome using BWA-mem2 (v2.2.1) [16], generating BAM (Binary Alignment Map) files. BAM files were then sorted and recalibrated using SAMtools (v1.18) [17] and Genome Analysis Toolkit (GATK; v4.4.0.0) [18], respectively. Variants were called using GATK (v4.4.0.0) for single nucleotide variants (SNVs) and small insertions/deletions (INDELs), 3bCNV (internally developed software) for copy number variants (CNVs), Manta (v1.6.0) [19] for structural variants (SVs), ExpansionHunter (v5.0.0) [20] for repeat expansions, GATK Mutect2 (v4.4.0.0) [18] for mitochondrial variant, and Mobile Element Locator Tool (MELT, v2.2.2) [14] for mobile element insertions (MEIs). Aneuploidy was predicted based on depth-of-coverage (DOC) information, and regions of homozygosity (ROH) were detected using AutoMap (v1.2) [21].

Various sequencing quality control metrics, such as the percentage of the genome covered at a minimum 20x DOC, the total number of variants, the heterozygous/homozygous variant ratio, the ti/tv (transition/transversion) variant ratio, and freemix for assessing potential contamination, were evaluated and confirmed to have passed before proceeding with downstream analyses (Table S1).

### 2.3. Variant Prioritization by EVIDENCE

Variants were annotated, filtered, and prioritized using EVIDENCE, an automated variant prioritization system which integrates a database module updated daily, a customized variant classification module, and a symptom similarity scoring module [22]. Following annotation by the Variant Effect Predictor (VEP) [23] using the most current databases, variants with allele frequencies exceeding 5% in the Genome Aggregation Database (gnomAD; v3.1; https://gnomad.broadinstitute.org/) [24] were excluded unless previously identified as pathogenic (P) or likely pathogenic (LP) in ClinVar (https://www.ncbi.nlm.nih.gov/clinvar/) [25] or the Human Gene Mutation Database (HGMD) [26] Professional.

Subsequently, variants were categorized as P, LP, variant of uncertain significance (VUS), likely benign (LB), or benign (B) in accordance with the American College of Medical Genetics and Genomics (ACMG) and American Molecular Pathology (AMP) variant classification guidelines [27], which were customized internally. Three in silico tools were employed to predict the pathogenicity of missense variants and the potential of a variant to alter splicing patterns: the rare exome variant ensemble learner (REVEL) [28], 3Cnet [29], and SpliceAI [30]. A symptom similarity score was then calculated for each gene in which a variant was found. Adapting the method described by Köhler et al. [31], the depth of Human Phenotype Ontology (HPO; https://hpo.jax.org/app/) [32] terms was used instead of information content to assess how similar the patient's symptoms are to the known symptoms of the disease associated with each gene. For repeat expansions, a variant was deemed P if the repeat number equaled or exceeded the normal range reported in the literature and the Online Mendelian Inheritance in Man (OMIM; https://www.omim.org/) [33] database. MEIs identified by MELT were considered potentially disease-causing if they occurred within a coding exon or an intronic region associated with the disease.

A list of rare variants was manually reviewed by the medical geneticists to select reportable variants. All reportable variants were manually reviewed in the Integrative Genomics Viewer (IGV; v2.9.4) [34] for technical validity. Clinical pathologists and clinical geneticists reviewed the final candidate variants.

### 2.4. Variant Confirmation and Family Testing

Except for SVs with exact breakpoints not identifiable by GS, all diagnostic P/LP variants were confirmed by orthogonal testing before reporting. For single nucleotide variants/small insertion or deletion variants (SNV/INDEL) and SV with exact breakpoints identified, Sanger sequencing was performed. Genomic DNA was extracted from whole blood using the QIAamp DNA Blood Mini Kit (Qiagen, Hilden, Germany). PCR primers (Macrogen, Seoul, Korea) were designed using Primer3 (v0.4.0; http://bioinfo.ut.ee/primer3-0.4.0/) [35, 36] and the National Center for Biotechnology Information (NCBI) GenBank (https://www.ncbi.nlm.nih.gov/genbank/) [37] reference sequence. PCR amplification and Sanger sequencing were performed using the PCR Master Mix Kit (Thermo Fisher Scientific, Waltham, MA, United States) and the SeqStudio Genetic Analyzer (Applied Biosystems, Foster City, CA, United States). The sequencing results were manually analyzed using Sequence Scanner Version 1.0 (Applied Biosystems).

For confirmation of the *SMN1* and *SMN2* gene variants, gene dosage assays were performed using primers (Bioneer, Daejeon, Korea) and MGB (minor groove binder) probes (Thermo Fisher Scientific) for *SMN1* and *SMN2* with RNaseP references. Amplification was done by the PerfeCta Multiplex qPCR ToughMix Low ROX kit (Quanta Biosciences, Gaithersburg, MD, United States) using the ABI 7500 system (Applied Biosystems). Multiplex ligation-dependent probe amplification (MLPA) analysis was performed using SALSA MLPA Probemix P021 SMA, P140 HBA, and P313 CREBBP (MRC Holland, Amsterdam, the Netherlands) according to the recommendations of the manufacturer. The PCR products were analyzed using a 3500 ABI sequencer (Applied Biosystems). Data analyses were done using the Coffalyser software (MRC Holland).

Complementary DNA (cDNA) sequencing was performed to confirm the expected splicing change caused by an intronic variant. Total RNA extraction was performed on an EDTA whole blood sample using QIAamp blood RNA (Qiagen), followed by cDNA synthesis using QuantiTect Reverse Transcription (Qiagen). Sanger sequencing was performed as described above.

Confirmation of SV without identifiable breakpoints was delegated to each regional center by recommending CMA/MLPA/karyotyping/FISH. Family testing was exclusively performed by an orthogonal test.

### 2.5. Variant Reporting

The GS test results were positive, negative, or inconclusive. A report was positive if one or more P/LP variants were identified as disease-causing and negative if there were no reportable variants. A report was inconclusive if (1) a heterozygous or hemizygous VUS was found in an autosomal dominant (AD) or X-linked (XL) disease gene; (2) biallelic variants were found, with at least one being a VUS in an autosomal recessive (AR) gene; or (3) a single P/LP variant was found in an AR gene. P/LP/US variants in a gene not associated with a disease in OMIM yet, but reported in the literature with sufficient evidence, were also categorized as inconclusive. The final result was shared with the healthcare providers through the portal.

### 2.6. Satisfaction Survey

A satisfaction survey was conducted online among regional center heads and vice heads, excluding lead and deputy centers. Out of 20 invited individuals, 15 (75%) responded. Survey items covered (1) satisfaction with results, (2) test reliability, (3) benefit to clinical management based on results, (4) patient satisfaction with the program, (5) convenience of the referral process, and (6) interest in continuing the program in the future.

### 2.7. Statistical Analysis

The confidence interval (CI) for the diagnostic rate (DR) was calculated using GraphPad online (https://www.graphpad.com/quickcalcs/confInterval1/). The chi-square test of independence was used to calculate the significance of the DR difference between males and females, and one-way ANOVA was performed to calculate the significance of the DR among different age-of-onset groups and across the regional centers using Microsoft Excel (Microsoft, Redmond, WA, United States).

## 3. Results

### 3.1. Participant Demographics

A total of 400 participants were enrolled in the pilot study. The male-to-female ratio was 1.26:1 (*n* = 223, Table S2). Most of the participants (88%, *n* = 351) had neonatal/infancy or childhood-onset, with the median age of onset being 1 year (mean: 4.39 years, standard deviation (SD): 6.71 years, Table S2). The median age at enrollment was 7 years (mean: 11.4 years, SD: 12.8 years), resulting in a delay of ~6 years between disease onset and genomic testing. A total of 240 (60%) participants were enrolled within 5 years of disease onset, 67 within 6–10 years, 44 within 11–15 years, and 49 after more than 15 years. All but 10 participants were of Korean descent.

Of 400 participants, 290 (72.5%) had prior genetic testing performed, with either negative or inconclusive results. All but 13 had fewer than three prior tests, with karyotyping (*n* = 128) being the most performed (Table S2). Notably, 12 participants had ES or GS performed, with negative or inconclusive results, and three of them were diagnosed through this study.

**Figure 1:**
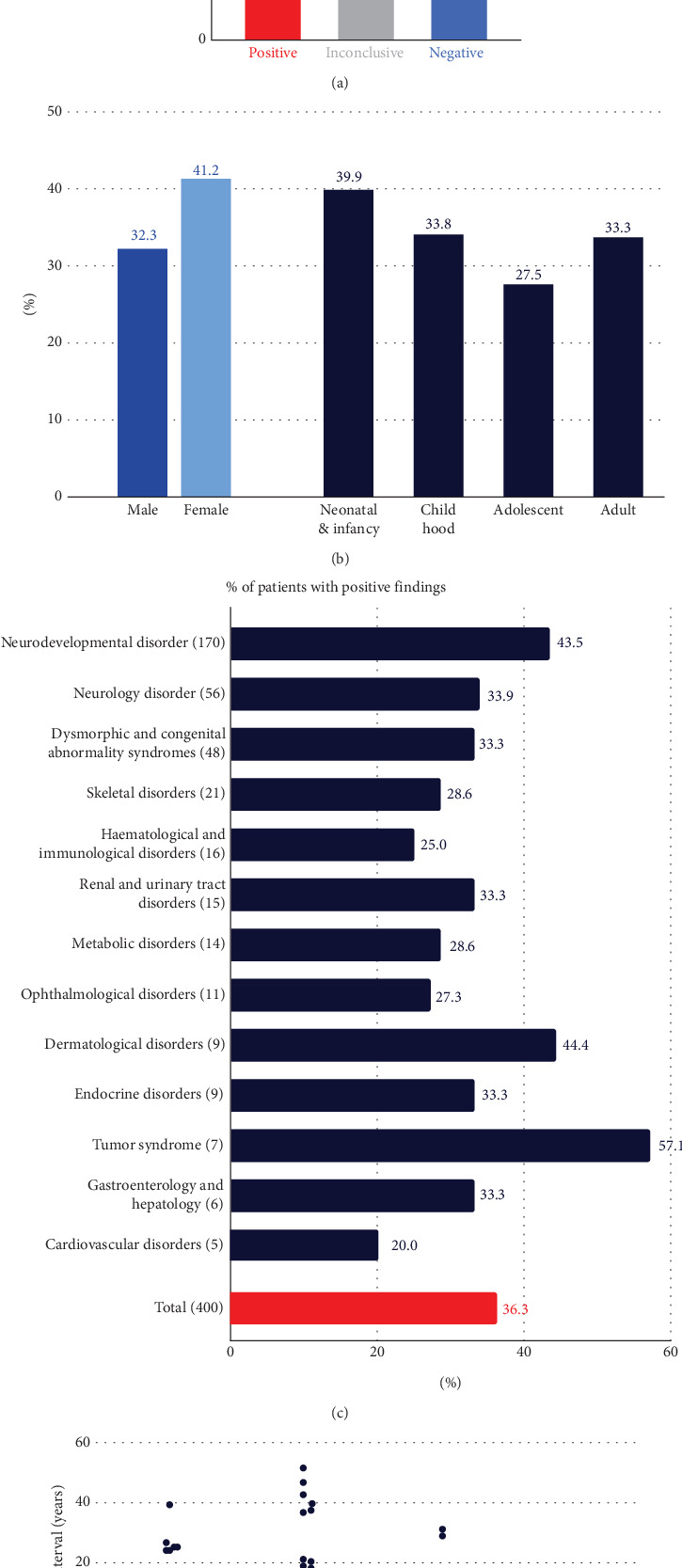
Diagnostic yield and test statistics. (a) The final diagnostic rate of the project. (b) Diagnostic rate by sex and age of onset. (c) Diagnostic rate by disease symptom categories. The number of participants for each category is noted in parentheses. (d) Interval in the year between the disease onset and enrollment to KR-RDSP.

The largest disease category of participants was neurodevelopmental disorders (*n* = 170), followed by neurology disorders (*n* = 56) and dysmorphic and congenital abnormality syndromic disorders (*n* = 48) (Figure 1(c)). The five most common symptoms referred for were global developmental delay (*n* = 117), epilepsy (*n* = 76), intellectual disability (*n* = 63), short stature (*n* = 44), and speech delay (*n* = 36).

### 3.2. Diagnostic Yield and Test Statistics

The final DR was 36.3%, with P/LP variants identified and reported as diagnostic (145/400, 95% CI: 31.7%–41.1%). An additional 43 participants had inconclusive findings (10.8%), and the rest (*n* = 212, 53%) received a negative result (Figure 1(a)). Of the 43 participants with inconclusive findings, 27 were reported with a heterozygous VUS in AD disease genes, eight with single heterozygous P/LP variants in AR disease genes, and five with compound heterozygous, with at least one being a VUS. The remaining three had either a low heteroplasmic mitochondrial LP variant, a heterozygous LP variant that only explains a subset of the symptoms, or an LP variant in a gene not associated with a disease in OMIM yet but reported in the literature.

The DRs were not significantly different by sex (*p* value: 0.20), age of onset (*p* value: 0.53), or regional centers (*p* value: 0.08) (Figure 1(b) and Figure S3). The DR varied by disease categories, ranging from 20% to 57.1% (Figure 1(c)). The neurodevelopmental disorder group, the largest group with 170 participants, had a DR of 42.5% (74/170, 95% CI: 36.3%–51.1%). The tumor syndrome group had the highest DR rate of 57.1% (4/7, 95% CI: 25.0%–84.0%), although it was the smallest group. The median age of this group was 17 years, suggesting that the younger participants were more likely to have cancer susceptibility gene variants than older participants with cancer [38]. The only disease category with more than five regional centers referring at least 10 participants was the neurodevelopmental disorder group, and the DR for this group ranged from 34.6% to 68.4%. However, the difference was not significant (*p* value: 0.38).

The 145 participants with positive findings were diagnosed with 115 different rare diseases. Of these, 13 were recurring diagnoses in 43 participants (Table S3). In 40 cases, a specific differential diagnosis was made based on the clinical phenotype before GS testing. In 30 of these cases, the disease-causing variants were identified in the suspected gene, while in the rest of the cases, a different disease gene was diagnosed.

For 145 participants with positive findings, it took a median of 4 years from disease onset to molecular diagnosis. For seven participants, the time to diagnosis exceeded 30 years (Figure 1(d)). The average TAT for GS testing was 29.6 days, with a maximum of 35 days (Figure S4).

### 3.3. Variant Reclassification by Family Testing

**Figure 2:**
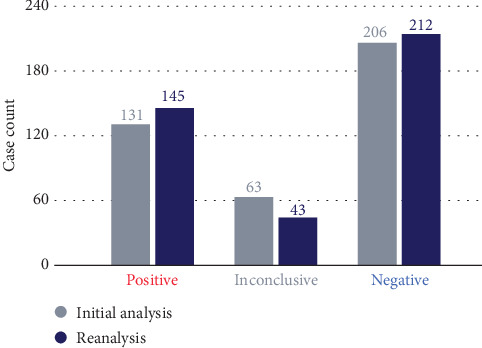
Changes in test results after family testing and reanalysis. Dark blue bars and light gray bars represent the number of participants in each category from the initial analysis and reanalysis after family testing, respectively.

Family testing of 120 probands from 61 families was performed for 78 variants (Table S4). Variants were determined to be assumed de novo in 15 probands, in *trans* in 12 probands, inherited from a similarly affected parent in 14 probands, inherited from an unaffected parent in two probands likely because of incomplete penetrance, inherited from an unaffected carrier mother on the X chromosome, and inherited from an unaffected parent in six probands. The variants were segregated, as expected, for five participants with at least one affected sibling tested (Table S5). Based on this added inheritance information, 15 reports changed from inconclusive to positive, five changed from inconclusive to negative, and one changed from positive to negative because the variant in a gene established as having complete penetrance was inherited from an unaffected parent (Figure 2).

### 3.4. Variant Characteristics

Of the 145 participants diagnosed with P/LP variants, 107 (73.8%) had SNV/INDEL in the nuclear genome, and five (3.4%) had an SNV in the mitochondrial genome. SVs were identified in 35 cases (24.1%): 28 with CNVs, one with repeat expansion, and one with an MEI. Fourteen variants could have only been identified by GS: five deep intronic variants, one untranslated region (UTR) variant, two mitochondrial SNVs of low (1.14% and 3.73%) heteroplasmic level, three CNVs spanning fewer than three consecutive exons and < 50 kb in size, two translocations, and one heterozygous deletion variant within a homologous sequence region (Table 1). The heterozygous deletion spanning a homologous *HBA1*/*HBA2* genic region would have been difficult to identify by ES. With GS, however, even though the exact breakpoints could not be identified, we could see clear evidence of a coverage drop in the intronic region. This variant was confirmed by MLPA.

An illustrative case is an 18-year-old female participant referred for suspected CHARGE (coloboma, heart defects, atresia, growth retardation, genital abnormalities, and ear abnormalities) syndrome with classic symptoms. Despite prior karyotyping, CMA, and even ES, a diagnosis remained elusive until GS revealed a heterozygous 0.3 kb deletion spanning Exons 30–31 of *CHD7*, associated with “CHARGE syndrome (OMIM: 214800)” that was suspected from the beginning. This molecular diagnosis, achieved 17 years after the onset of symptoms, illustrates how the disease-causing variant can be missed even when the correct disease is suspected because of an inappropriate test method.

**Figure 3:**
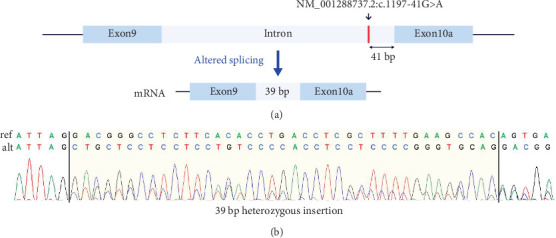
The predicted splicing alteration was confirmed by cDNA sequencing. (a) A deep intronic variant near Exon 10a of the *DNM1* gene was predicted to result in acceptor loss and acceptor gain by SpliceAI, resulting in an exon extension of 39 bp. (b) Sanger sequencing traces showing the heterozygous 39 bp insertion in the cDNA.

Another case is an 8-year-old male participant with a suspected neurodegenerative disorder. He had seizures starting at age 1 year, followed by developmental delay, severe intellectual disability, walking difficulty, facial features, and vision impairments. GS identified a heterozygous deep intronic VUS in the MANE (Matched Annotation from the NCBI and EMBL-EBI) transcript of *DNM1*, associated with AD “developmental and epileptic encephalopathy 31A (OMIM: 616346).” However, this variant is close to an exon in the brain-specific transcript (NM_001288737.2) that is more relevant to this disease than the MANE transcript [39]. The variant, located near a well-established intronic variant that adds two amino acids to Exon 10a [39], was confirmed by cDNA sequencing to extend Exon 10a by 13 amino acids as predicted (Figure 3).

### 3.5. Secondary Findings

All participants opted in to receive secondary findings in the 81 genes selected by the ACMG for being medically actionable [40]. Six participants received known or expected P variants in the following genes: *PMS2*, *MYL3*, *MAX*, *TTN*, *MYBPC3*, *SCN5A*, and *PCSK9*. One person had variants in two genes.

### 3.6. Satisfaction Survey

All respondents found the test results reasonable, with 88.8% expressing satisfaction. All respondents found the test results acceptable, with 90% indicating it to be reliable. All respondents agreed the test results were beneficial for clinical management and that participants were satisfied. Regarding the referral process, 77.7% found it easy. Finally, all respondents emphasized the importance of continuing the program (Table S6).

## 4. Discussion

With support from the Division of Rare Disease Management of the KDCA for 8 months, the KR-RDSP implemented a centralized genomic testing workflow for 11 regional rare disease centers to efficiently perform GS and molecularly diagnose patients with suspected rare genetic disorders. The pilot study of 400 participants demonstrated satisfactory DRs across all regional centers, with a comparable overall DR to published data [41, 42]. As expected, GS facilitated the detection of various types of genetic variants beyond SNV/INDEL, highlighting its effectiveness in identifying the genetic cause. The implementation of a centralized workflow of GS testing involving a CAP (College of American Pathologists) and CLIA (Clinical Laboratory Improvement Amendments) accredited laboratory was beneficial in allowing stringent quality control and tight monitoring of the TAT.

Even though trio sequencing that analyzes the parental samples simultaneously is reported to have a higher DR in general [43] because it allows the identification of de novo and compound heterozygous variants, here, a proband-only approach was taken to maximize the use of resources and test as many participants as possible. Having a large internal database, of which the majority is Korean, that we could use as controls in addition to the gnomAD database [24] helped remove variants that were rarely or not observed in gnomAD but high internally and more likely to be B. Sanger sequencing was used for family testing, which yielded a high rate (31.7%) of reclassification from VUS to LP or LB. Another reason Sanger sequencing could fit seamlessly into the workflow was that all diagnostic variants had to be Sanger confirmed regardless of family testing. With Next-Generation Sequencing (NGS) technology proving to be highly accurate across most of the genomic regions, it has become a common practice to perform Sanger confirmation only on the variants with low quality [44, 45]. However, in Korea, ES and GS-based tests are still considered “research,” and therefore, ES/GS reports are not accepted for the insurance purpose of follow-up tests, while Sanger sequencing reports are. More initiatives like this program will be able to prove the utility of ES/GS tests and advocate the need to approve genomic testing as diagnostic in Korea.

Of the diagnosed participants who had prior genetic testing, seven participants had seven variants reported as P/LP by the current study that were already reported from the previous testing. Two variants previously reported as VUS were classified as LP. The difference was likely due to how each laboratory customizes the ACMG variant classification guidelines. Three variants previously reported as VUS were classified as LP after family testing, which was probably not pursued before because there were too many VUS in the prior reports, while from the current study, only variants considered most likely were reported and recommended for family testing. Finally, there were two participants with a variant already reported as LP or P from a prior test but considered not diagnostic because of a likely unclear clinical correlation. Globally, the rates of inconclusive VUS being reported from panel testing are high [46], and it could impose a burden on clinicians who, in general, do not have the means to follow up. As seen in our cases, the clinicians may opt to order another test, as they cannot determine which variants to test further. Therefore, not only is an accurate variant interpretation important but also a clear report of the diagnostic finding is essential. There were no exclusion criteria for enrollment. However, in the continuing studies, we could potentially impose exclusion criteria for participants with a likely answer from prior testing by more rigorously screening centrally.

As the study was not conducted in parallel with the conventional workflow of performing genomic testing for the regional patients, the diagnostic findings, yield, and TAT are not direct comparisons. However, based on the comparable diagnostic yield to other GS studies and the satisfactory survey results showing the study exceeding the expectations of the regional centers, we can speculate that the diagnostic yield was not compromised while shortening the diagnostic odyssey by relieving the traveling burden for the patients.

Forty-three cases remain inconclusive even after family testing. A subset of the 27 participants who have not undergone family testing yet could potentially receive updated positive results after family testing. For eight participants with a single heterozygous P/LP variant in an AR disease gene, there may be a second variant not identifiable by short-read NGS technology or not interpretable at the time. This applies to participants with negative results as well. Further studies, such as RNA sequencing or long-range sequencing, may resolve some of the VUS or negative cases, and reanalyzing the GS data routinely may yield diagnoses for some of the participants.

Finally, an important practical value of genomic testing for rare disease patients lies in its potential financial benefits. By obtaining an accurate diagnosis and a predictive understanding of the disease course, patients and their families can reduce unnecessary medical expenses and gain access to financial assistance for medical care. While the reduction in medical costs is difficult to quantify in a short-term study, we can estimate the number of patients who could benefit from the “special exemption for rare diseases” program provided by the Korean National Health Insurance Service (NHIS) [47]. Typically, once a patient receives a genetic diagnosis associated with a qualifying “special exemption” code, the NHIS covers 90% of their medical expenses, significantly reducing their financial burden. In this study, 83 patients were diagnosed with a condition linked to such a code, making them eligible for financial assistance. For the remaining 62 patients diagnosed with a gene or disease not yet associated with a corresponding code, their physicians can apply to establish a new code. These programs not only alleviate the financial strain on individual patients and families but also promote a sense of solidarity and shared responsibility, ultimately strengthening the broader community.

## 5. Conclusions

This study demonstrated the utility and feasibility of implementing a centralized genetic testing workflow for regional patients in Korea and the effectiveness of GS for diagnosing patients with suspected rare diseases. As a result of this success, the program has been approved to continue in the following year.

## Figures and Tables

**Table 1:** Characteristics of P/LP variants and variants that were only identifiable by genome sequencing.

**Variant types**	**No. of variants**
SNV/INDEL (nuclear genome)	107
SNV/INDEL (mitochondrial genome)	5
Repeat expansion	1
Mobile element insertion	1
Aneuploidy	2
Large structural variants including copy number variants	31
Total	145

**Variants only identifiable by genome sequencing**	

**Variant types**	**No. of variants**
Deep intronic SNV/INDEL	5
Untranslated region SNV/INDEL	1
Low heteroplasmic mitochondrial variant	2
< 3 consecutive and < 50 kb exonic deletion/duplication	3
Translocation	2
Variant in homologous sequence	1

**Variant classification**	**No. of variants**
Pathogenic	4
Likely pathogenic	5
Variant of uncertain significance	5

**Report conclusions**	**No. of participants**
Positive	7
Inconclusive	7

Abbreviation: SNV/INDEL, single nucleotide variant/small insertion and deletion.

## Data Availability

The data that support the findings of this study are available from the corresponding authors upon reasonable request. The data are not publicly available due to privacy or ethical restrictions.

## Supporting Information

Supporting Information Additional supporting information can be found online in the Supporting Information section. Figure S1: The KR-RDSP workflow. Figure S2: Genome sequencing test workflow. Table S1: Sequencing quality metrics statistics. Table S2: Patient demographics. Figure S3: The diagnostic rate across the regional centers. Table S3: Recurrent genes reported as diagnostic. Figure S4: Distribution of the turnaround time. Table S4: Results from family testing. Table S5: Summary of the families tested. Table S6: Satisfaction survey summary.
