# Supplementary material for: Genome Sequencing of Rare Disease Patients Through the Korean Regional Rare Disease Diagnostic Support Program
Source: Hum Mutat. 2025 Feb 27;2025:6096758. doi: 10.1155/humu/6096758 (PMC11987077; doi:10.1155/humu/6096758)
Supplement: Supporting Information — Additional supporting information can be found online in the Supporting Information section. Figure S1: The KR-RDSP workflow. Figure S2: Genome sequencing test workflow. Table S1: Sequencing quality metrics statistics. Table S2: Patient demographics. Figure S3: The diagnostic rate across the regional centers. Table S3: Recurrent genes reported as diagnostic. Figure S4: Distribution of the turnaround time. Table S4: Results from family testing. Table S5: Summary of the families tested. Table S6: Satisfaction survey summary. [file 6096758.f1.docx]

**Supplementary Information**

**Figure S1. The KR-RDSP workflow**

**Figure S2. Genome sequencing test workflow**

**Table S1. Sequencing quality metrics statistics**

**Table S2. Patient demographics**

**Figure S3. The diagnostic rate across the regional centers**

**Table S3. Recurrent genes reported as diagnostic**

**Figure S4. Distribution of the turnaround time**

**Table S4. Results from family testing**

**Table S5. Summary of the families tested**

**Table S6. Satisfaction survey summary**


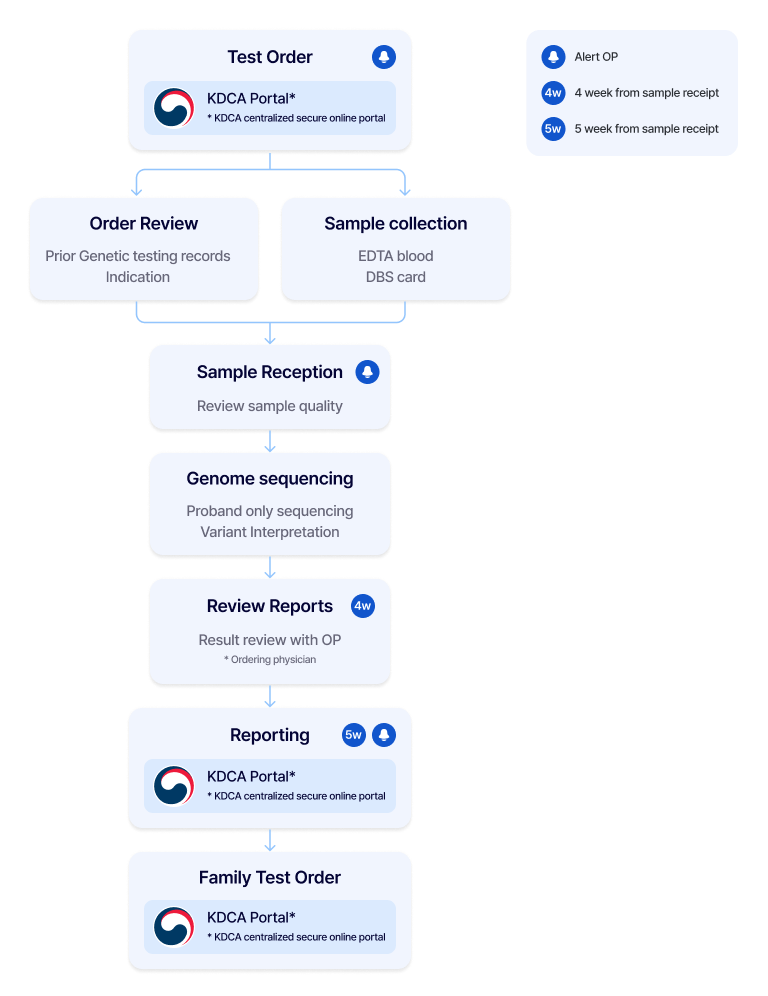


**Figure S1. The KR-RDSP workflow.** The entire workflow of the KR-RDSP was managed within the centralized secure online portal. Notifications were automatically sent to the clinicians when samples were received by the laboratory, and reports were uploaded to the portal.

**
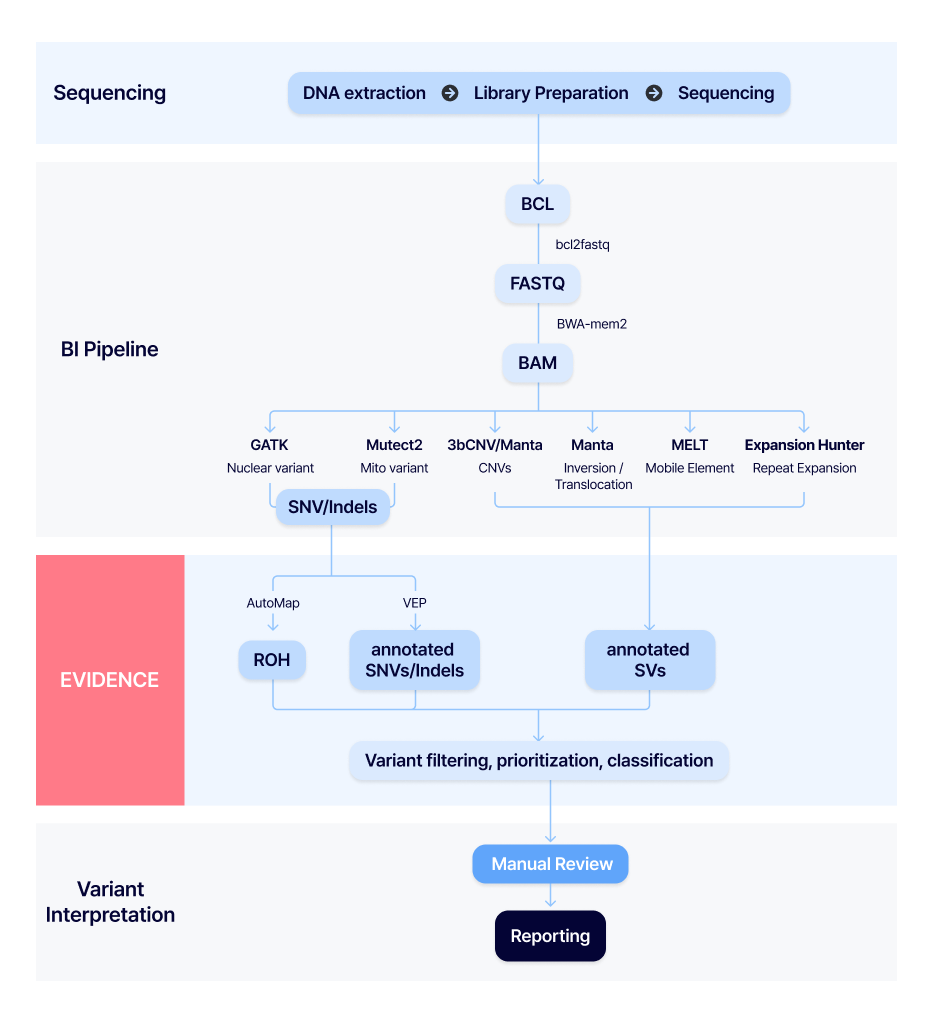
**

**Figure S2. Genome sequencing test workflow.** Workflow of the GS pipeline from sample receipt to reporting of the test results at the centralized GS testing laboratory.

**Table S1. Sequencing quality metrics statistics**

| Parameters | Acceptable ranges | Results (Mean) |
| --- | --- | --- |
| Q30 | >85% | 91.80% |
| Mean depth | >30x | 44.91x |
| % of the genome covered at >= 20x | >95% | 97.18% |
| Total number of SNVs | 3,800,000-4,700,000 | 4,157,953 |
| Total number of small INDELs | 900,000-1,200,000 | 1,020,732 |
| Transition/transversion variant ratio | 1.90-1.95 | 1.92 |
| Heterozygous/homozygous variant ratio | 0.9-2.3 | 1.4 |
| Freemix | <0.02 | 0.0005 |

**Table S2. Patient demographics**

|  | No. patients |
| --- | --- |
| Sex (male:female) | 223:177 |
| Age of onset | No. patients |
| Neonatal & infancy (< 2) | 213 |
| Childhood (2-11) | 138 |
| Adolescent (12-18) | 40 |
| Adult (≥19) | 9 |
| Mean age of onset | 4.4 years |
| Mean age at enrolling | 11.4 years |
| Time interval between disease onset and enrollment | No. patients |
| 0 years | 98 |
| 1-5 years | 142 |
| 6-10 years | 67 |
| 11-15 years | 44 |
| ≥16 years | 49 |
| Mean (SD) interval | 7 (9.8) years |
| No. diagnostic tests performed before enrollment | No. patients |
| 0 | 110 |
| 1 | 174 |
| 2 | 72 |
| 3 | 34 |
| 4 | 7 |
| ≥5 | 3 |
| Type of tests performed before enrollment | No. patients |
| Karyotyping | 128 |
| Chromosome microarray | 112 |
| Single gene test | 52 |
| NGS panel test | 127 |
| ES/GS | 12 |
| Other | 23 |

NGS, next-generation sequencing; ES. Exome sequencing; GS, genome sequencing, No, numbers; SD, standard deviation


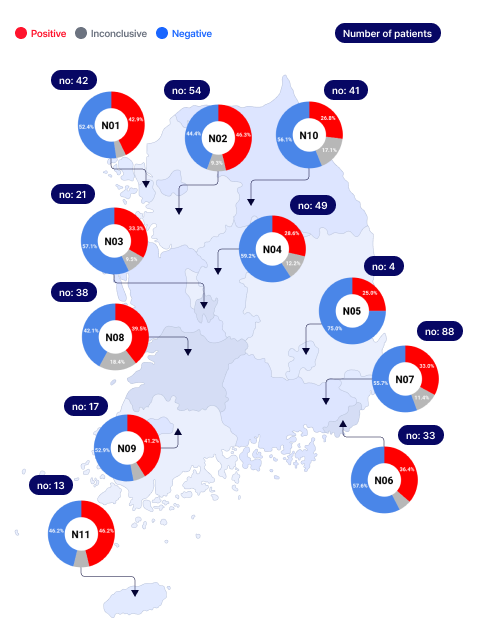


**Figure S3. The diagnostic rate across the regional centers.** The locations of each regional center and their final diagnostic rates are shown The total number of patients referred from each center is indicated.

**N01**: Inha University Hospital, **N02**: Ajou University Hospital, **N03**: Chungnam National University Hospital, **N04**: Chungbuk National University Hospital, **N05**: Kyungpook National University Chilgok Hospital, **N06**: Inje Univ Busan Paik Hospital, **N07**: Pusan National University Yangsan Hospital, **N08**: Jeonbuk National University Hospital, **N09**: Chonnam National University Hwasun Hospital, **N10**: Yonsei University Wonju Severance Christian Hospital, **N11**: Jeju National University Hospital

**Table S3. Recurrent genes reported as diagnostic**

| Gene (Disease) | No. occurrences |
| --- | --- |
| *NF1* (Neurofibromatosis, type 1) | 10 |
| *PTEN* (Cowden syndrome 1) | 10 |
| *PTPN11* (Noonan syndrome 1) | 3 |
| *GNAS* (Pseudohypoparathyroidism Ia) | 2 |
| *NSD1* (Sotos syndrome) | 2 |
| *NUS1* (Intellectual developmental disorder, autosomal dominant 55, with seizures) | 2 |
| *SLCO2A1* (Hypertrophic osteoarthropathy, primary, autosomal recessive 2) | 2 |
| *CACNA1A* (Episodic ataxia, type 2) | 2 |
| *CHD7* (CHARGE syndrome) | 2 |
| *MT-ND1* (Leber optic atrophy and dystonia) | 2 |
| *ERF* (Craniosynostosis 4) | 2 |
| *SMN1* (Spinal muscular atrophy) | 2 |
| *TSC2* (Tuberous sclerosis-2) | 2 |

**
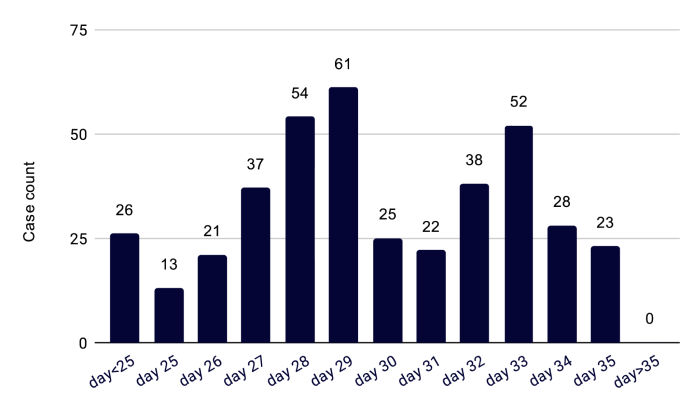
**

**Figure S4. Distribution of the turnaround time.** Distribution of the turnaround time (TAT) for the entire study cohort of 400 probands. Time spent on family testing was not included. The X-axis is the TAT in days and the Y-axis is number of patients.

**Table S4. Results from family testing**

| Number of families (subjects) tested | 61 (120) |
| --- | --- |
| Cases with changes in test results by family testing | No. patients |
| Inconclusive -> Positive | 15 |
| Inconclusive -> Negative | 5 |
| Positive -> Negative | 1 |
| Total | 21 |
| Distribution of inheritance | No. patients |
| Assumed *de novo* | 15 |
| Confirmed in *trans* | 12 |
| Confirmed in *cis* | 1 |
| inherited from a (mildly) affected parent | 14 |
| inherited from an unaffected parent | 4 |
| Inherited from a parent with an uncertain phenotype | 2 |
| Shared with similarly affected family member(s) | 1 |
| Not shared with similarly affected family member(s) | 1 |
| Total | 50 |
| Variants reclassified by family testing | No. variants |
| LP -> P | 6 |
| VUS -> LP | 15 |
| VUS -> LB | 7 |
| LP -> LB | 1 |
| Total | 29 |
| Positive reports of affected members by family testing | 10 |

LP, Likely pathogenic; P, pathogenic; LB, likely benign; VUS, variant of uncertain significance

**Table S5. Summary of the families tested**

| Disease inheritance | both parents tested | single parent tested | only sibling tested | total |
| --- | --- | --- | --- | --- |
| AD | 35 | 4* | 1 (unaffected) | 40 |
| AR | 14 |  | 2 (both affected) | 16 |
| XL | 1 | 2 |  | 3 |
| MT |  | 1 | 1 (affected) | 2 |
| total | 50 | 7 | 4 | 61 |

***** Two were affected mothers, 1 was an unaffected mother, and 1 was an unaffected mother with an unaffected sibling and an affected sibling

**Table S6. Satisfaction survey summary**

|  | Strongly  agree | Agree | Neutral | Disagree | Strongly disagree |
| --- | --- | --- | --- | --- | --- |
| Results were satisfactory | 44.4% | 44.4% | 11.1% | 0% | 0% |
| Testing was reliable | 60% | 30% | 10% | 0% | 0% |
| Results were helpful for clinical management | 50% | 50% | 0% | 0% | 0% |
| The referral process was convenient | 22.2% | 44.4% | 33.3% | 0% | 0% |
| The program needs to be continued | 80% | 20% | 0% | 0% | 0% |
| The patients were satisfied with the results | 40% | 60% | 0% | 0% | 0% |
